# Serum tissue polypeptide-specific antigen (TPS): what is its diagnostic value?

**DOI:** 10.1054/bjoc.1999.1219

**Published:** 2000-04-27

**Authors:** D Valik, M Nekulova

**Affiliations:** Department of Biochemistry, Masaryk Memorial Cancer Institute, Zluty kopec 7, Brno, 656 53, The Czech Republic


in the text. Dr Kirkwood suggests that there are a number of 'errors' in the editorial, but provides no evidence for these, although there is a difference in our interpretations of the *available* data, which is incomplete. In the absence of the definitive report on E1690 it is reasonable to *speculate* that stage migration and changes in surgical technique might explain the difference between the results and those of E1684. Dr Kirkwood points out one such change in that twice as many patients had clinically negative unresected lymphatics in the later study. Only he can appreciate the role, if any, of sentinel node biopsy until the E1690 results are published.

The editorial acknowledges that overall survival was improved by high-dose interferon (HDI) in the original study. Amongst the four sub-groups analysed in the trial report only patients with clinically apparent lymphadenopathy showed a statistically significant improvement in survival. It is fair to say that the group with clinically negative histologically positive nodes was too small to allow interpretation of interferon's efficacy. However, to maintain that this is the population with the most to gain from HDI on the basis of a 34 patient sample is tenuous, and not a claim Dr Kirkwood made in the original report on E1684.

We are agreed that HDI is active in melanoma, and that cross-over salvage therapy is the most plausible explanation for the conflicting results obtained. Given its toxicity and the suggestion that it remains effective at second relapse further work is required to pinpoint the role of HDI in melanoma. Thus, it is not possible to commend HDI as the standard adjuvant therapy in melanoma at high risk of recurrence. Indeed, various cooperative groups are pursuing trials in this field in which the control arm is observation only and Schering Plough recently abandoned their study in resected stage III melanoma in which HDI was the control arm.

Patients should undoubtedly be informed of the results of both trials, but only in the USA will they be permitted to accept or reject treatment. The conflicting results of the E1684 and E1690 studies mean that few purchasers in the UK currently funds HDI for melanoma at high risk of recurrence. Only patients with the means to fund treatment themselves will be able to come to their own decision. The way forward is to design and execute studies that address the issues thrown up by the imminent publication of the full E1690 results.

*MR Middleton*
*CRC Department of Medical Oncology,*
*Christie Hospital NHS Trust, Wilmslow Road,*
*Withington, Manchester M20 4BX, UK*

DOI: 10.1054/ bjoc.1999.1219, available online at http://www.idealibrary.com on IDEAL®

### Serum tissue polypeptide-specific antigen (TPS): what is its diagnostic value?

**Sir**,
We have read with interest an article of Rebhandl et al (1998) on the diagnostic usefulness of the tissue polypeptide-specific antigen (TPS) in neuroblastoma and Wilms tumour. We would like to share our clinical experience with TPS, which is less convincing than that presented, and have a comment to add on the theoretical and technical part of the paper.

Traditionally, TPA – summing fragments of (cyto)keratins 8, 18, 19 – and TPS – the soluble fragment of (cyto)keratin 18 – have been interpreted by some researchers as markers for cell proliferation (Einarsson and Rylander, 1997; Mishaeli et al, 1998). With the advent of knowledge on apoptosis it has been found that one of the central effector molecules, caspase-3, utilizes (cyto)keratin 18 but not (cyto)keratin 8 as a substrate (Caulin et al, 1997). This recent observation implies that (cyto)keratin 18 may be specifically degraded upon receiving an apoptotic stimulus, thus putatively producing a TPS-like material. We currently explore this concept on the MCF-7 breast cancer-derived cell line, which is deficient in caspase-3 (Janicke et al, 1998). Altogether, tumour markers based on detection of (cyto)keratin fragments, TPA, TPS and CYFRA21-1 may, at least to some extent, reflect degradative rather than proliferative cellular events. Apoptosis-inducing antitumour therapy (cytotoxic drugs, radiotherapy) leads to downstream activation of caspase-3 in most systems studied (Hannun, 1997). The question of cleavage products of this reaction with (cyto)keratin 18 as a substrate has not yet been addressed.

In the presence of sepsis and/or renal insufficiency, TPS values are indeed elevated. However, minor or localized infection and liver and/or multiorgan failure can also lead to elevation of TPS with either no apparent underlying malignancy or no change in stable disease, as we have repeatedly observed in our patients. The authors state, that 'these samples were, therefore, excluded' without giving specific criteria. It should be noted that TPA/TPS are fairly unspecific biomarkers and for diagnosis are of similar value as erythocyte sedimentation rate. We assume that diagnoses in these patients were based on standard techniques. In this sense, interpreting TPS as a diagnostic marker and assessing its specificity using ROC after careful *a priori* elimination of confounders seems inappropriate, since the TPS value apparently adds nothing to the diagnostic procedure. On the other hand, data from Table 1 of the paper indicate that TPS could be interpreted as a therapy response marker (Pronk et al, 1997) as long as variables (intercurrent infections, etc.) are under control. It would also be informative to include comparison with established biomarkers for neuroblastoma and Wilms' tumour (catecholamines, NSE). That 'the potential of TPA in Wilms' tumour (Ishiwata et al, 1991) have gone unnoticed in the literature', as the authors state, may merely reflect the fact that the TPA value has never contributed new and clinically relevant information.

At our institute, we performed measurement of TPA for about 8 years (approx. 5500 measurements year$^{-1}$); 7 months ago we replaced TPA with TPS Beki (Sweden) due to automation and

 © 2000 Cancer Research Campaign avoidance of radioactivity. We used both mostly as disease recurrence markers for adult patients with solid tumours, mainly breast and colorectal cancers, believing, as others, that this reflects the proliferative status of the particular tumour (Nekulova et al, 1995; Van Dalen et al, 1998; Nisman et al, 1998). We have abandoned the practice of monitoring patients with TPS (ASCO, 1997) after 7 months when we had completed 2703 tests. During this time we observed isolated elevation of TPS higher than 140 U $l^{-1}$, in 61 patients (breast and colorectal cancer), with other relevant markers (CA15-3, CEA, CA19-9) being below cutoff. In only four of them recurrence of disease was confirmed (breast cancer assessed by CA15-3, CEA, imaging techniques and complete examination by a medical oncologist). In two other patients (breast cancer) a suspect finding appeared on bone gallium scan, which was not subsequently confirmed on CT scan. Another two patients presenting with elevation of TPS were classified as stable disease (breast cancer, local partial remission). In another 55 patients restaging was performed at the discretion of a medical oncologist in charge, however, without contributing new information.

Especially data on TPS elevations approaching up to 2600 U $l^{-1}$ with no apparent disease progression in patients otherwise classified as ‘complete remission’ added significant stress to patients and their doctors and generated substantial unnecessary testing. Although inconclusive at this point, this group of false positives suffered mostly from chronic inflammatory and/or noninflammatory skin affections (herpetic infections, unhealed defects after radiotherapy with or without secondary bacterial infections and in some the reason was not apparent). In clinical practice the true difference between ‘false positivity’ and ‘lead time’ is difficult to distinguish during a limited time period; those markers with lead times longer than 7 months do not prove very useful for influencing patient outcome – this may obviously be the case of some of our 55 patients. It is our belief that the validity of new biomarkers and reevaluation of those used previously (Robertson, 1998) should be critically reassessed on a periodic basis if the ultimate goal is to improve patient care while avoiding unnecessary increases in noise and cost of health care. In agreement with the authors of the present paper, we currently recognize the potential of serum TPS only as a marker for monitoring response to cytotoxic therapy in explicitly defined diagnostic groups and when the potential of administering curative therapy exists, which indeed may include neuroblastoma and Wilms’ tumor. In our opinion, TPS should not be used as a diagnostic marker and only in exceptional cases as a marker for disease recurrence.

*D Valik, M Nekulova*
*Department of Biochemistry,*
*Masaryk Memorial Cancer Institute,*
*Zluty kopec 7, 656 53, Brno, The Czech Republic*
Supported in part by the Scientific and Research Scheme MZ00020980501.

### REFERENCES

ASCO (1997) Update of recommendations for the use of tumor markers in breast and colorectal cancer. Adopted on November 7, 1997 by the American Society of Clinical Oncology. *J Clin Oncol* **16(2)**: 793–795

Caulin C, Salvesen GS and Oshima RG (1997) Caspase cleavage of keratin 18 and reorganization of intermediate filaments during epithelial cell apoptosis. *J Cell Biol* **138(6)**: 1379–1394

Einarsson R and Rylander L (1997) Tissue polypeptide-specific antigen (TPS) detects a specific epitope structure on human cytokeratin. *Anticancer Res* **17**: 3121–3124

Hannun YA (1997) Apoptosis and the dilemma of cancer chemotherapy. *Blood* **89(6)**: 1845–1853

Ishiwata I, Ono I, Ishiwata C, Soma M, Nakaguchi T, Ohara K, Hirano M and Ichikawa H (1991) Carcinoembryonic proteins produced by Wilms tumor cells in vitro and in vivo. *Experimental Pathology* **41**: 1–9

Janicke RU, Sprengart ML, Wati MR and Porter AG (1998) Caspase-3 is required for DNA fragmentation and morphological changes associated with apoptosis. *J Biol Chem* **273(16)**: 9357–9360

Mishaeli M, Klein B, Sadikov E, Bayer I, Koren R, Gal R, Rakowsky E, Levin I, Kfir B, Schachter J and Klein T (1998) Initial TPS serum level as an indicator of relapse and survival in colorectal cancer. *Anticancer Res* **18(3B)**: 2101–2105

Nekulova M, Pecen L, Eben K, Simickova M and Slama M (1995) Prediction of disease recurrence in oncological patients. *Neural Network World* **6(5)**: 945–950

Nisman B, Lafair J, Heching N, Lyass O, Baras M, Peretz T and Barak V (1998) Evaluation of tissue polypeptide specific antigen, CYFRA 21-1, and carcinoembryonic antigen in non-small cell lung carcinoma: does the combined use of cytokeratin markers give any additional information? *Cancer* **82(10)**: 1850–1859

Pronk LC, Stoter G, van Putten WL and de Wit R (1997) The correlation of CA15-3 and TPS with tumor course in patients with metastatic breast cancer. *J Cancer Res Clin Oncol* **123(2)**: 128–132

Rebhandl W, Rami B, Turnbull J, Felberbauer FX, Paya K, Bancher-Todesca D, Gherardini R, Mittlboeck M and Horcher E (1998) Diagnostic value of tissue polypeptide-specific antigen (TPS) in neuroblastoma and Wilms tumor. *Br J Cancer* **78(11)**: 1503–1506

Robertson JFR (1998) Blood tumour markers in breast cancer. *Tumour Marker Update* **10**: 31–37

Van Dalen A, Barak V, Cremaschi A, Gion M, Molina R, Namer M, Stieber P, Sturgeon C and Einarsson R (1998) The prognostic significance of increasing marker levels in metastatic breast cancer patients with clinically complete remission, partial remission or stable disease. *Int J Biol Markers* **13(1)**: 10–15

DOI: 10.1054/ bjoc.1999.1245, available online at http://www.idealibrary.com on IDEAL®

### Response to: Serum tissue polypetide-specific antigen (TPS): what is its diagnostic value – reply

**Sir**,

We are glad that our paper (Rebhandl et al, 1998) has aroused the interest of Drs Valik and Nekulova and that they can agree with our conclusions regarding the potential of TPS as a tool for monitoring therapy response. However, we would like to make a few comments on this letter.

First of all, our paper did not report clinical experience. The situation in paediatric oncology is very different from adult oncology. Apart from catecholamines and NSE in neuroblastoma (not in Wilms’ tumor) there are no ‘established’ tumour-markers. For TPS we actually had to establish normal values for healthy children (Rebhandl et al, 1997) before addressing patients with malignant disease. Furthermore, all our data are based on TPS and not on TPA, which in our opinion is not comparable.

Breast and colorectal cancer are among the most frequent malignant diseases in the western world, while the incidence of

© 2000 Cancer Research Campaign

